# Photosynthetic Responses of *Anthurium* × ‘Red’ under Different Light Conditions

**DOI:** 10.3390/plants10050857

**Published:** 2021-04-23

**Authors:** Lingyan Chen, Muhammad Waqqas Khan Tarin, Heqiang Huo, Yushan Zheng, Jianjun Chen

**Affiliations:** 1 College of Landscape Architecture, Fujian Agriculture and Forestry University, Fuzhou, Fujian 350002, China; fafucly@fafu.edu.cn (L.C.); waqas_tarin@yahoo.com (M.W.K.T.); 2 Mid-Florida Research Education Center and Environmental Horticulture Department, Institute of Food and Agricultural Sciences, University of Florida, Apopka, FL 32703, USA; hhuo@ufl.edu

**Keywords:** plant metabolism, photosynthesis, light acclimatization, chlorophyll

## Abstract

Light is an essential energy source for plant photosynthesis, although it can also be a stress-causing element. Therefore, the current research was aimed to compare photosynthetic responses of *Anthurium* × ‘Red’ leaves at different positions (bottom old leaf, 1; center mature leaf, 2; top expanded leaf, 3) established under three photosynthetic photon flux densities (PPFDs): 550 μmol·m^−2^·s^−1^ as high (H), 350 μmol·m^−2^·s^−1^ as medium (M), and 255 μmol·m^−2^·s^−1^ as low (L). After six months, all the replicates were relocated to interior rooms with a PPFD of 30 μmol·m^−2^·s^−1^. There were no significant differences in chlorophyll concentration of the old leaf among treatments, before (Day 0) and after shifting the plants to interior rooms (Day 30). The total chlorophyll concentrations of the mature and top leaves increased significantly. In greenhouse conditions, H and M treatments did not show any significant change for net photosynthetic rate (*P*n) at various leaf positions. However, M2 exhibited an improved *P*n in the interior conditions. Plants grown under M treatment were greener and had bigger leaves compared to other treatments. Our study reveals that *Anthurium* × ‘Red’ photosynthesis responses to different light conditions varied distinctly. However, M treatment can keep the plants looking green by accumulating enough energy for indoor conditions, and middle and lower leaves may be triggered to restore photosynthetic activity under low light or indoor conditions.

## 1. Introduction

Shading is widely employed during the growth of plants, particularly plants with ornamental foliage to prevent damage triggered by high light intensity [1,2]. When light-harvesting antennas absorb more light energy than their potential for photochemical and non-photochemical energy dissipation, photodamage can occur [3]. In the most severe cases, this may result in the discoloration of leaves or necrosis. Light damage happens often as a result of continuous exposure to high light intensity levels [4]. As a result, growers apply shades to foliage or shade plants by covering the shutter or using a whitewash on the greenhouse cover to avoid direct exposure under high light conditions.

Light is an essential energy source for plant photosynthesis, although it can cause photodamage [5]. Light is a key environmental factor that influences the morphological and physiological performance of plants. Plants that are exposed to a specific irradiance are mostly adapted to this light environment [6]. For instance, plants exhibit notable adaptability and plasticity to varying light conditions by modifying their photosynthetic apparatus and morphological traits [5]. Various indoor, foliage plants are exposed to low-light environments for a long time after being sold [1]. The plants with a high photosynthesis ability under low light or shade conditions acclimate naturally to survive by decreasing their light compensation points and increasing leaf size and chlorophyll contents [7,8].

The net photosynthetic rate/chloroplastic CO_2_ response curve (*P*n/*C*c curve) and the *P*n/*I* curve are effective measures in plant physiology. Both curves help researchers to consider the consequences of differences in one or more major elements causing photosynthesis [9]. Photosynthesis is a key physiological trait to assess the general performance and photosynthesis ability of plants [10]. It is also known as the assimilation rate, which is an important physiological index to determine the growth efficiency of plants. Photosynthesis in plants can be influenced by various factors, such as leaf age and position, sink effects, mutual shading, as well as environmental factors, such as light, temperature, nutrition, and water availability [11]. Therefore, we attempt to acquire insight into the potential of leaves at various positions, developed and matured under different shade levels to conform to the complexity of their photosynthetic response.

Studies suggest that fully expanded leaves have been used to determine the net photosynthetic rate (*P*n) in plants [12,13]. However, few previous studies have also compared the photosynthesis between newly emerged leaves and fully developed mature leaves. For instance, in wild-type tobacco plants, newly emerged leaves have the lowest *P*n compared to that of fully developed mature leaves [14]. Furthermore, the application of nitrogen (N) fertilization at different concentrations did not influence the net photosynthetic rate and carboxylation rate (Vc_max_) of the incomplete leaves. However, under various treatments, the *P*n and Vc_max_ of the incomplete leaves varied significantly [15]. In the process of continuous differentiation and development of new leaf tissues of plants, N storage plays an important role in the synthesis of photosynthetic proteins [16] and continues to differentiate until the leaf stops expanding. Therefore, fully developed leaves are representative of the indicative photosynthetic capacity in plants [17,18].

The fully expanded leaves in plants can be categorized as, young, mature, and old leaves. Evidence suggests that during their development stages, the *P*n also varies. For example, under high light conditions, the *P*n of the third leaf of wheat plants reached a maximum on the seventh day after emergence and declined thereafter [19]. Zhou et al. [20] compared the temperature responses of photosynthesis and respiration of both the young and old leaves of *Quercus aquifolioides* in an alpine oak forest, where the old leaves have shown a lower net assimilation rate relative to the young leaves. However, the fully expanded leaves of kiwifruit have exhibited lower respiration rates compared to young leaves [21]. In addition, leaf position is also related to light interception, which may influence the CO_2_ assimilation [22]. Escalona et al. [23] have demonstrated that Spanish grapevine leaves exhibit comparable radiation use efficiency from all locations of the canopy except for those in the central part, although other considerations, such as different leaf age might play only a minimal role. However, contradictory results have been reported for Asian pear leaves, where nodes (3 to 16) have greater saturation vapor pressure and transpiration rates. Both the apical and basal leaves have higher stomatal resistance and lower *P*n than the leaves located in an intermediate position [24]. The aforesaid studies did not document reliable photosynthetic responses of ornamental plants owing to their leaf age, position, and expanded conditions under varying light conditions. Therefore, the complexity of these plant’s photosynthesis and their adaptability under different shade levels require further attention.

Plants from the genus *Anthurium* are popular ornamental foliage plants [25,26]. *Anthurium* × ‘Red’ is widely used as an indoor ornamental plant. It has a long flowering period as well as bright green leaves and is renowned for its high aesthetic value. *Anthurium* × ‘Red’can be adaptive to shade conditions and has a long life as an indoor plant. We hypothesized that different light levels would impact not only the photosynthetic activity of *Anthurium* × ‘Red’ plants but also their ornamental quality and subsequent performance under interior conditions. To test this hypothesis, the current research was designed to investigate the variations in photosynthesis of *Anthurium* × ‘Red’ under different light conditions, to compare their photosynthetic potential at different leaf positions and ages, and to evaluate their performance after being placed in interior conditions. It was anticipated that such a study could provide growers and interior plantscapers with science-based information on better production and indoor use of *Anthurium*, and foliage plants in general.

## 2. Results

### 2.1. Changes in Net Photosynthetic Rate of Anthurium × ’Red’ before and after Placement in Interior Rooms from Greenhouse

**Figure 1:**
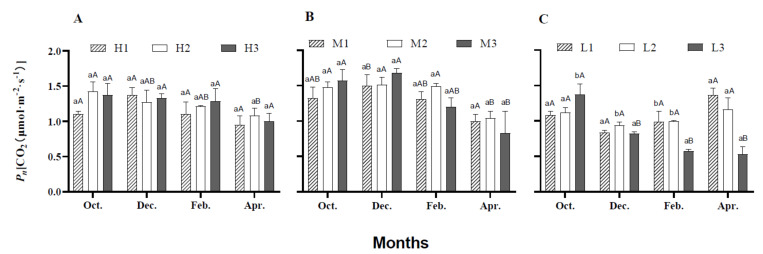
Net photosynthetic rate [CO_2_ (μmol·m^−2^·s^−1^)] of *Anthurium* × ‘Red’ in greenhouse conditions. Where H (**A**), M (**B**), and L (**C**) represent PPFDs at 550, 350, and 255 μmol·m^−2^·s^−1^, respectively. Data are presented as means, and vertical lines at each bar are the standard errors. Different lowercase letters indicate significant differences among different leaf positions in the same month, while uppercase letters indicate significant differences among various months at the same position based on Tukey HSD (honestly significant difference) test at *P <* 0.05 level. Numeric values 1, 2, and 3 represent the bottom old leaf, center mature leaf, and the top young expanded leaf, respectively.

During the six months of greenhouse growth, the *P*n varied among the three shade levels in different months (Figure 1). H treatment (550 μmol·m^−2^·s^−1^) did not show any significant change in *P*n at various leaf positions (1, 2, and 3 represent the bottom old leaf, center mature leaf, and the top young expanded leaf, respectively) within each month. The *P*n was not significantly influenced in different months at the same leaf positions for the plants established under H treatment. However, in October, the *P*n of H2 was significantly (*P <* 0.05) higher compared to that in April (Figure 1A).

Under M treatment (350 μmol·m^−2^·s^−1^), no significant differences were noticed among the three leaf positions within the same month. In December, a significantly (*P <* 0.05) greater *P*n was recorded compared to that in April for M1. For M2, *P*n decreased significantly in April compared to that in other months, and the same trend was observed for M3 (Figure 1B).

In October, under L treatment (255 μmol·m^−2^·s^−1^), L3 showed a significantly higher *P*n compared to that of L1 and L2. Whereas in December, L2 responded with higher (*P <* 0.05) *P*n relative to that of L1 and L3. In February and April, the *P*n of L3 decreased significantly (*P <* 0.05) relative to that of L1 and L3 (Figure 1C).

**Figure 2:**
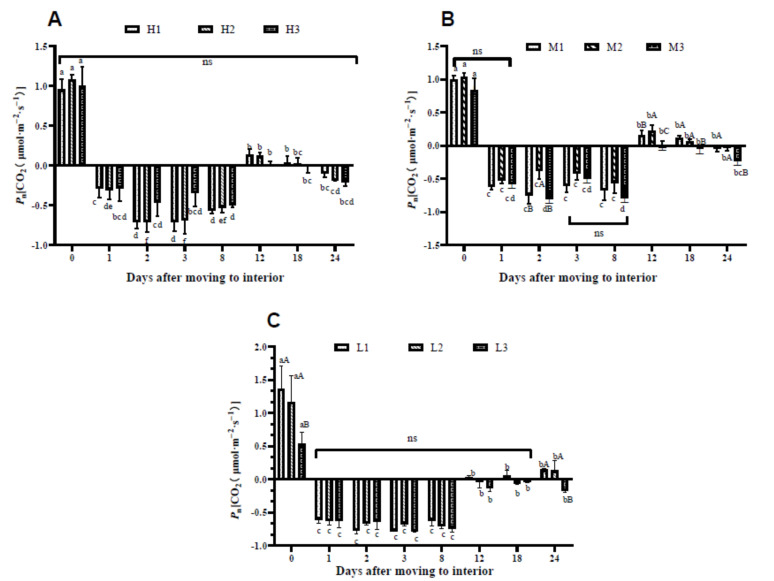
Net photosynthetic rate [CO_2_ (μmol·m^−2^·s^−1^) of *Anthurium* × ‘Red’ after placement in interior rooms. Where H (**A**), M (**B**), and L (**C**) represent PPFDs at 550, 350, and 255 μmol·m^−2^·s^−1^, respectively. The numeric values 1, 2, and 3 represent the bottom old leaf, center mature leaf, and the top young expanded leaf, respectively. Vertical lines at each bar are the standard errors, and ns represent the non-significant differences at three leaf positions Different lowercase and uppercase letters represent significant differences among leaves in tested days and leaf positions on the same day, respectively, based on Tukey HSD test at *P <* 0.05 level.

After moving into the interior rooms with a PPFD of 30 μmol·m^−2^·s^−1^, the *P*n values of all the plants dropped to negative (Figure 2). On the first day, the *P*n values were negative and continued to be negative until the 12th day. However, the *P*n started to recover on the 12th day after being relocated to the interior rooms. All the marked leaves under three treatments exhibited a very low *P*n value from the 12th day to the 18th day, then became negative again on the 24th day except for L1 and L2. In terms of various leaf positions within each day, in the plants established under H treatment, we found no significant differences (Figure 2A). Under M treatments, M2 showed significantly higher *P*n values over those of M1 and M3 on the 12th day after moving to the interior room (Figure 2B). However, for the plants established under the L treatments, *P*n at three leaf positions did not show any differences from the 1st to the 18th day. However, from the 12th day, the plants restored their *P*n and then became stable; close to 0 CO_2_ (μmol·m^−2^·s^−1^), particularly the *P*n of L1 and L2 increased slowly. On the 24th day, the *P*n values of L3 were significantly lower than those of L1 and L2 (Figure 2C).

### 2.2. Light–Response Curves of Photosynthesis of Anthurium × ‘Red’ before and after Placement in Interior Rooms

The photosynthetic parameters of the leaves under different light conditions in the greenhouse are depicted in Table 1. Where α is the initial slope of the photosynthesis curve, which indicates light use efficiency (LUE) in the low light condition. Under H treatment, α values for the three positioned leaves showed no significant differences. In plants under M treatment, α values of M1 were significantly lower than those in M2 and M3. However, for plants grown under L treatment, α values had a significant trend of L2 > L1 > L3. Plant’s *P*n-max under H treatments showed that H3 was significantly higher than H1 and H2; similarly, under M treatment M3 was significantly higher than M2 and M1. However, for the plants established under L treatments, *P*n-max for L1 was significantly higher than that of L2 and L3. It suggested that the young leaves on the top had a stronger photosynthetic ability under H and M treatments, while bottom old leaves (L1) had a stronger photosynthetic ability than L2 and L3 when grown under the low light level. Overall, leaf position influenced the *P*n-max of *Anthurium* (Table 1). Moreover, *I*sat (the saturation irradiance) of M3 was significantly higher than that of other treatments. *I*c (light compensation point) decreased when the light level decreased as well as when the leaf position declined, especially under H and M treatments. The *I*c levels of top new leaves were significantly higher than those of middle mature and bottom old leaves, but under L treatment, the differences in *I*c between three positions were not significant. According to the differences of the α value, *P*n-max, and *I*c, among the leaves of different positions under various treatments, it appeared that the new leaves of plants under M treatment showed better photosynthetic ability. While the leaves around the bottom parts showed a higher photosynthesis ability than the top and middle parts under L treatment.

After moving the plants to the interior rooms, compared to the greenhouse conditions, the *P*n-max and *I*sat values were lower, but *I*c values were higher (Table 1 and Table 2). Compared with different leaf positions under each treatment, H2 and M2 showed significantly higher *P*n-max values than other positioned leaves under H and M treatment, respectively, but under L treatment, L1 and L2 showed a significantly higher *P*n-max than that of L3 indicating the middle mature leaf under H and M treatment played a major role in photosynthesis. While under L treatment, the bottom old and middle mature leaves showed a higher photosynthetic ability than the top leaf, which is similar to the greenhouse condition.

Results of two-way ANOVA revealed that in the greenhouse, the light condition, leaf position, and their interactions significantly (*P <* 0.05) influenced the α. Only leaf position significantly impacted both the *P*n-max (*P <* 0.05) and *I*sat (*P <* 0.01), whereas light condition and its interaction with leaf position did not show any influence. Furthermore, leaf position and light condition both affected the *I*c, but their interaction was not significant (Table 3). However, in interior conditions, leaf position and its interaction with light condition influenced both the α (*P <* 0.05) and *I*sat (*P <* 0.01) significantly. Light condition, leaf position, and their interaction also significantly impacted the *P*n-max (*P <* 0.05) as depicted in Table 3.

**Figure 3:**
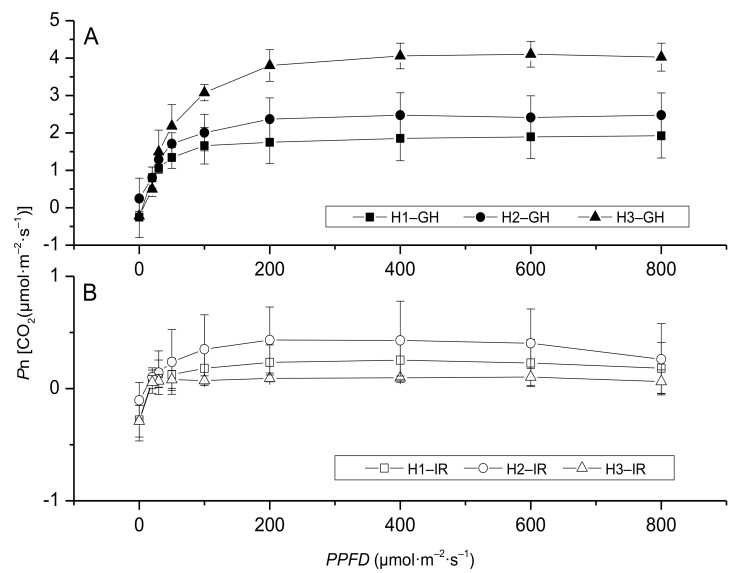
Light–response curves of *Anthurium* × ‘Red’ grown in a shaded greenhouse under a high light level (550 μmol·m^−2^·s^−1^) before (**A**) and after (**B**) placement to interior rooms. Where H: high light level, IR: interior room, and numeric values 1, 2, and 3 represent the bottom old leaf, center mature leaf, and the top young expanded leaf, respectively.

According to the light—response curve of photosynthesis, the result showed significant differences among leaves at different positions. In the greenhouse high light conditions, the top new leaves (H3) had a higher *P*n than the leaves in other positions (H1 and H2), i.e., the top expanded leaves were largely responsible for improved photosynthetic activity (Figure 3A). After the plants moved to the interior rooms, the three photosynthetic fitting curves did not show any difference, all the *P*n values stayed at low levels. However, center mature leaves (H2) had a higher *P*n value than that of H1 and H3, which means the center mature leaves played an increasing role in photosynthesis (Figure 3B).

**Figure 4:**
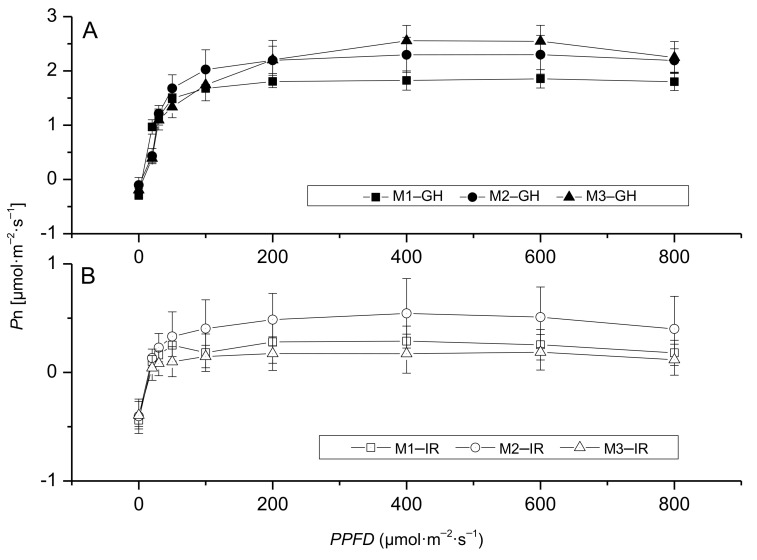
Response of net photosynthetic rate of *Anthurium* × ‘Red’ grown under a medium light level (350 μmol·m^−2^·s^−1^) before (**A**) and after (**B**) placement to interior rooms. Where M: medium light level, IR: interior room, and numeric values 1, 2, and 3 represent the bottom old leaf, center mature leaf, and the top young expanded leaf, respectively.

The plants established under M treatment showed different light—response curves under greenhouse and interior conditions. In the greenhouse condition, different from H treatment, the light—response curves of leaves from different positions were staggered and overlapped (Figure 4A). After moving to interior conditions, similar to H treatment, M2 showed greater *P*n values compared to the other two (M1 and M3), which means the mature leaves at the middle position escalated the photosynthesis for the plants under interior conditions (Figure 4B; Table 2).

**Figure 5:**
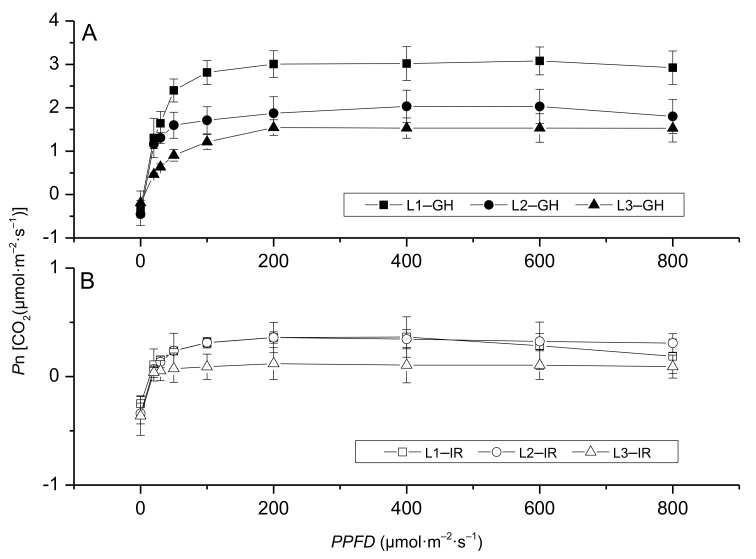
Response of net photosynthetic rate of *Anthurium* × ‘Red’ grown under a low light level (255 μmol·m^−2^·s^−1^) before (**A**) and after (**B**) placement to interior rooms. Where L: low light level, IR: interior room, and numeric values 1, 2, and 3 represent the bottom old leaf, center mature leaf, and the top young expanded leaf, respectively.

The plants established in the greenhouse conditions under L treatment showed varied photosynthetic light–response curves for leaves at different positions. The *P*n values of L1 were higher than those for L2 and L3 (Figure 5A; Table 1), suggesting that the bottom old leaf had a better performance in photosynthesis under L treatment in the greenhouse. However, in the interior room, the *P*n was not larger than that at 0.5 μmol·m^−2^·s^−1^ (Figure 5B). According to the photosynthetic parameters and the light response curve of plants under L treatment in the interior rooms, L1 and L2 showed a better photosynthesis performance than L3, which shows that the new top leaves hardly contribute to the energy accumulation in the interior conditions (Table 2).

### 2.3. Chlorophyll Concentration of Anthurium × ‘Red’ Leaves before and after Placement in Interior Rooms

In greenhouse conditions, the leaf’s color established under M and L treatments was dark-green (more fit to the requirement of horticulture products) whereas the leaf color of plants under H treatment was yellow-green with sun scorch. For the first leaf under all treatments, there was no significant difference in chlorophyll concentration before (Day 0) and after moving the plants to interior rooms (Day 30). However, the chlorophyll concentrations of both the second and third leaves of all the three treatments (H, M, and L) increased significantly (*P <* 0.05) since they moved to the interior room (Day 0 to 30; Table 4 and Table 5).

### 2.4. Changes of Leaf and Flower Condition between Shaded Greenhouse and Interior Conditions

Table 6 compares the leaf number, flower counts, and longevity and growth index between the plants before (greenhouse) and after moving to the interior room. The results revealed compared to greenhouse conditions, that plants established under M and L treatment had better flower longevity after moving to the interior room, in which the flower longevity of the M treatment was better. However, H treatment did not show any difference from greenhouse conditions to the interior room, but because of a high amount of accumulated energy, the flower had a long performance time.

### 2.5. Appearances of Anthurium × ‘Red’ under Different Light Condition

**Figure 6:**
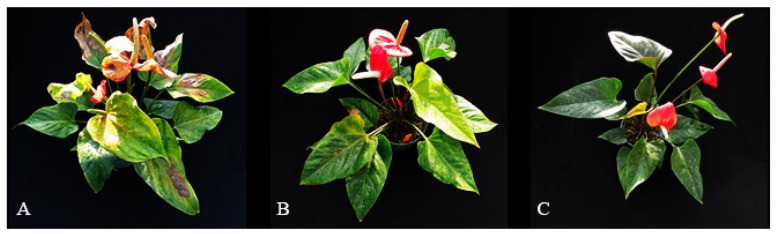
The appearances of *Anthurium* × ‘Red’ grown in a shaded greenhouse under H (**A**), M (**B**), and L (**C**) light levels (550, 350, and 255 μmol·m^−2^·s^−1^, respectively).

Morphologically, *Anthurium × ‘Red’* plants under H treatments showed yellowish spots, which turned to brownish (burning) in the lateral stage (Figure 6A). Whereas plants under M treatment were comparatively greenish and had larger leaves than other treatments (Figure 6B). However, under L treatments, the leaves were darker in color with a smaller size (Figure 6C).

## 3. Discussion

### 3.1. Anthurium Showed Different Performance under Different Light Conditions

In the present study, *Anthurium* × ‘Red’ showed divergent adaptability under various light intensities. When the plants were exposed to the high light (H) initially, the chlorophyll contents decreased to prevent photoinhibition and light burn or sunburn, whereas, under low light level (L), chlorophyll contents increased to improve the light use efficiency. Variation in chlorophyll concentrations is thought to be an ubiquitous trend in plants, they are indices of plant metabolism and are primarily assessed by the availability of nutrients and ecological factors [27]. Moreover, it is a well-known phenomenon for shade-tolerant species to increase chlorophyll contents with decreasing irradiance to facilitate light-harvesting [28,29]. Under high light conditions, chl-b degradation is induced by an isozyme; [30], while it increases under low light [31]. Secondly, the compensation irradiance decreased to improve the photosynthesis ability of plants under L treatments. Thirdly, plants reactivate the photosynthesis ability of older leaves to adapt to low light conditions.

Leaves at different positions exhibited different photosynthetic responses to production light levels. The role of the middle and top leaves of *Anthurium* × ‘Red’ was significant to regulate photosynthesis. Compared to the H treatment, *P*n-max, *I*sat, and *I*c values of the leaves in the middle and on the top positions decreased under L treatment. However, the older leaves in the bottom showed a higher *I*sat under L treatment relative to that under H treatment. Photosynthetic pigments in the mesophyll cells determine the color and photosynthetic function of the leaves [31]. Under low light, plants change their physiological characteristic to get more light energy, for example, increasing leaf chlorophyll contents [32]. However, under extreme light stress, the photosynthetic process is restricted, leading to leaves etiolating [33]. It has been reported previously that plants under low light were less productive than those under high light [34]. Compared with low light, excessive light generates harmful oxygen radicals that may give rise to the process of photoinhibition and reduction in the primary productivity of plants [35], which can explain the scorches on leaves of plants under H treatment. There have been few reports that medium light conditions (about 50% of full sunlight) led to higher levels of biomass production in few species [36], which is similar to the case for *Anthurium*. Furthermore, previous studies have indicated that under normal environments, plants have to adapt themselves in response to fluctuations in light intensity [37,38].

### 3.2. Appropriate Light Could Improve Anthurium × ‘Red’ Performance Indoors

Apart from the natural environment, indoor ornamental plants can adapt to stressful environments by different types of receptors, including photoreceptors [39]. Under the absence of light or in limited light conditions, plants develop etiolation symptoms, such as a decrease in chlorophyll contents, reduction in leaf area, or hypocotyl elongation [33]. In this experiment, the compensation irradiance of *Anthurium* × ‘Red’ was below 5 μmol·m^−2^·s^−1^ while the saturation irradiance range was from 347.40 to 483.96 μmol·m^−2^·s^−1^ (Table 1), and the PAR of the indoor condition was about 30 μmol·m^−2^·s^−1^, so the *P*n reduced greatly (Figure 3, Figure 4 and Figure 5) and influenced the plant growth. Nevertheless, the plants under different light treatments in greenhouse conditions showed a different response than those under interior conditions. Therefore, we conclude that *Anthurium* × ‘Red’ plants during the course of nursey production undergo a process of accumulating light energy. Plants under a suitable light condition accumulated light energy resulting in greater photosynthetic ability. After shifting the plant to interior conditions, the flower counts and longevity remained comparably appealing for 24 days. However, *P*n values of all marked leaves quickly dropped one day after placement indoors, recovered 12 days later, but became negative again on the 24th day except for L1 and L2. These results suggest that a long time under 30 μmol·m^−2^·s^−1^ indoors will decrease the photosynthesis ability of *Anthurium* × ‘Red’ and low-light acclimatization in the greenhouse can delay the process [1]. However, exposure to high light and high temperature should be avoided, which may affect plant quality by chlorosis and sunburn as evidenced in previous reports on other ornamental plants [38]. To improve the quality of indoor ornamental plants and extend the flowering time, light intensity must be adjusted properly during the production, and plants must be acclimatized under a low light level before placement indoors, which could improve the indoor performance of foliage plants [1].

### 3.3. Leaf Position Will Influence the Photosynthesis Character

Leaf position can affect photosynthesis and transpiration. In normal conditions, the *P*n of fully expanded mature leaf tends to be higher than that of the newly emerged leaf at the top and the descended leaf [11,40]. Quite the reverse, some plants show a different trend, such as kiwifruit, the expanded leaves of which had a lower *P*n [21]. Our findings indicated that leaves at the central position may better exploit low irradiance than young leaves on the top and the oldest leaves at the bottom, while the young leaves perform better under greenhouse conditions. A similar phenomenon was observed in our previous study with *Anthurium* ‘Red Hot’ [8] and also in grapevine [41]. It is obvious that the *P*n of leaves at different positions might show a significant difference, and leaf senescence will affect photosynthetic efficiency. Leaf photosynthesis can be influenced by stomatal conductance and intercellular carbon dioxide [10,42]. The relative importance of these two factors has been studied in several plants, which suggests that intercellular carbon dioxide is the predominant factor. As in the previous research, the stomatal conductance fluctuation was positively correlated with leaf age but no interaction was found with intercellular carbon dioxide [41]. In young and old leaves, lower soluble protein and chlorophyll concentrations per unit leaf area result in alleviation of *P*n [16,41,43]. Contradictory to previous findings, under L treatment in the greenhouse and interior conditions, *Anthurium* × ‘Red’ mature leaves at the middle and bottom position may be triggered to rejuvenate or regain photosynthetic activity [8]. Therefore, this mechanism needs further exploration.

## 4. Materials and Methods

### 4.1. Plant Materials and Growth Conditions

The current study was conducted in Central Florida from October 2018 to April 2019. Tissue-cultured liners of *Anthurium* × ‘Red’ were transplanted to 15 cm diameter containers (height = 30 cm and diameter = 15 cm) filled with Vergo Mix A. Each container was top-dressed with 5 g of an eight-month formulation of Osmocote 17-7-12 (The Scott Co., Marysville, OH, USA) and watered once a week. Fifteen plants with a similar growth size and leaf color were selected, divided into three groups, and grown in a shaded greenhouse under three light levels. The greenhouse was covered by double layer polyethylene film, and shade cloth with three different densities was installed inside, resulting in three sections with daily maximum PPFDs of 550, 350, and 255 μmol·m^−2^·s^−1^ as high (H), medium (M), and low (L) levels, respectively. The experiment was arranged as a completely randomized design with five replications. After plants were established in a shaded greenhouse for six months, three leaves were selected and marked as 1, 2, and 3 for a bottom old leaf, center mature leaf, and top young expanded leaf, respectively on each plant. We marked the leaves of H group as H1, H2, and H3, M group as M1, M2, and M3, and L group as L1, L2, and L3. Thereafter, all the replicates were moved to the interior room with a light intensity of 30 μmol·m^−2^·s^−1^,12 h a day, provided by white fluorescent lamps following Li et al. [2]. 

### 4.2. Leaf Greenness Estimated by SPAD

Leaf SPAD (Soil—Plant Analysis Development) readings of the marked leaves were recorded before and one-month after plants were moved to the interior rooms by using a SPAD-502 m (Konica-Minolta, Japan) as described by Wang et. al. [44]. Five independent SPAD measurements were determined on each marked leaf of each plant, and total chlorophyll concentrations were determined following Wang et al. [45].

### 4.3. Net Photosynthetic Rate Comparison of Anthurium × ‘Red’ under 30 PPFD

The *P*n was measured once every two months (10/15/2018, 12/18/2019, 2/15/2019, and 4/16/2019) during greenhouse growing as well as 1, 2, 3, 6, 8, 12, 18, and 24 days after moving to the interior room, respectively. All the measurements were carried out at a photosynthetic photon flux density (PPFD) value of 30 μmol·m^−2^·s^−1^, which was the same as the interior room light condition, a CO_2_ concentration of 400 μmol·mol^−1^ on a sunny day between local time 9:00 and 12:00 a.m. by using the Li-6800 portable photosynthesis system (LI-COR, Inc., Lincoln, NE, USA). Three leaves from different positions on each plant were measured and there were five plants in each group.

### 4.4. Light–Response Curve Comparisons of Anthurium× ‘Red’

For each treatment, three marked leaves were measured for light—response curves before (0) and 12 days after moving to the interior rooms, respectively. A photosynthetic photon flux density (PPFD) gradient of 0, 10, 20, 30, 50, 100, 200, 400, 600, and 800 μmol·m^−2^·s^−1^ and a CO_2_ concentration of 400 μmol·mol^−1^ were used for the measurement of irradiance responses using a Li-6800 portable photosynthesis system (LI-COR, Inc., Lincoln, NE, USA). A half an hour photoinduction under 300 μmol·m^−2^·s^−1^ was carried out before each measurement. The leaf temperature was 25 ± 0.5 °C, and the relative humidity was 50 ± 1%.

The irradiance (I)–response curves of photosynthesis were fitted following the modified model of the rectangular hyperbola [46] as follows:

(1) $$P\left(I\right)=\frac{\left(1-\beta I\right)}{\left(1+\gamma I\right)}\left(\alpha I+Rd\right)$$

where *P*(I) is *P*n; *R*d is the rate of dark respiration; and α, β, and γ are the coefficients that are independent of I.

The compensation irradiance, *I*c, was calculated as follows [47]:

(2) $$Ic=-\frac{Rd}{\alpha }$$

The saturation irradiance, *I*sat, was determined using the following formula [47]:

(3) $$I\mathrm{sat}=\frac{\sqrt{\left(\beta +\gamma \right)/\beta }-1}{\gamma }$$

The maximum photosynthetic rate, *P*n-max, was calculated as follows [47]:

(4) $$Pn-\max=\alpha \frac{\sqrt{\beta +\gamma }-\sqrt{\beta }}{\gamma }-Rd$$

### 4.5. Changes in Plant Morphology before and after Moving into the Interior Rooms

The number of leaves, newly emerged leaves, flower count, flower longevity, and growth index were also determined monthly for the first six months, then, all the plants were moved to interior rooms, where these attributes were recorded weekly.

### 4.6. Data Analysis

SPSS software (version 19.0, IBM Corp., Armonk, NY, USA) was used for the statistical analysis of the data. All data were subjected to analysis of variance (ANOVA). If significance occurred among treatments, means were separated by Tukey HSD (honestly significant difference) test at *P* < 0.05 level. All the values were presented as mean ± standard errors. Additionally, the software Origin^®^ v. 8.5 (Origin-Lab Corp., Northampton, MS, USA), Prism v. 8.0.1 (GraphPad, San Diego, CA, USA), and Microsoft Excel-2016 were used for visualization (light–response curve fitting model) and tables, respectively.

## 5. Conclusions

The purpose of this research was to investigate variations in photosynthesis of *Anthurium* × ‘Red’ under different light conditions and compared the photosynthetic potential of leaves at different positions during plant production and interiorscaping to document plant dynamic responses for adapting to different growing conditions. We conclude that plants grown under a medium light level (350 μmol·m^−2^·s^−1^) can maintain green-colored leaves and accumulate substantial energy for sustaining indoor growth. Furthermore, *Anthurium* × ‘Red’ has shown the ability to survive in low light conditions, which could be in part attributed to the rejuvenation of leaves at middle and bottom positions for enhanced photosynthesis.

## Figures and Tables

**Table 1:** Photosynthetic parameters of the leaves under different light conditions in the greenhouse.

Treatments	α	*P*n-Max [CO_2_ (μmol·m^−2^·s^−1^)]	*I*sat (μmol·m^−2^·s^−1^)	*I*c (μmol·m^−2^·s^−1^)	*R*d [CO_2_ (μmol·m^−2^·s^−1^)]	Adjusted R^2^
H1–GH	0.09 ± 0.02 c	1.88 ± 0.61 cd	355.31 ± 77.18 b	2.98 ± 1.01 ab	0.25 ± 0.21 a	0.987
H2–GH	0.09 ± 0.02 c	2.47 ± 0.61 bcd	359.13 ± 79.67 b	3.10 ± 0.80 ab	0.26 ± 0.20 a	0.994
H3–GH	0.08 ± 0.01 c	4.18 ± 0.33 a	459.94 ± 61.17 b	4.58 ± 0.81 a	0.35 ± 0.11 a	0.989
M1–GH	0.14 ± 0.02 b	1.85 ± 0.14 cd	391.59 ± 80.68 b	2.46 ± 0.09 b	0.30 ± 0.06 a	0.999
M2–GH	0.07 ± 0.02 c	2.37 ± 0.37 bcd	359.77 ± 83.03 b	2.77 ± 0.45 ab	0.19 ± 0.12 a	0.966
M3–GH	0.06 ± 0.01 c	3.48 ± 0.68 ab	483.96 ± 54.51 a	4.26 ± 0.02 ab	0.22 ± 0.01 a	0.982
L1–GH	0.15 ± 0.01 b	3.12 ± 0.52 abc	358.12 ± 70.87 b	2.45 ± 0.03 b	0.34 ± 0.23 a	0.997
L2–GH	0.20 ± 0.01 a	1.94 ± 0.34 cd	347.40 ± 55.83 b	2.74 ± 0.62 b	0.45 ± 0.02 a	0.991
L3–GH	0.05 ± 0.01 c	1.57 ± 0.36 d	366.23 ± 46.94 b	2.77 ± 0.83 ab	0.19 ± 0.04 a	0.994

Where H, M, and L represent PPFDs at 550, 350, and 255 μmol·m^−2^·s^−1^, respectively, and numeric values 1, 2, and 3 represent the bottom old leaf, center mature leaf, and the top young expanded leaf, respectively. Data are presented as means ± standard error (n = 5). Different letters represent significant differences among treatments based on Tukey HSD test at a *P* < 0.05 level.

**Table 2:** Photosynthetic parameters of the leaves under different light conditions in the interior rooms.

Treatments	α	*P*n-Max [CO_2_ (μmol·m^−2^·s^−1^)]	*I*sat (μmol·m^−2^·s^−1^)	*I*c (μmol·m^−2^·s^−1^)	*R*d [CO_2_ (μmol·m^−2^·s^−1^)]	Adjusted R^2^
H1–IR	0.05 ± 0.01 cd	0.23 ± 0.02 cd	291.95 ± 30.24 a	3.33 ± 0.13 a	0.28 ± 0.15 a	0.9813
H2–IR	0.01 ± 0.01 e	0.45 ± 0.04 a	272.89 ± 50.14 a	2.78 ± 0.55 cd	0.10 ± 0.09 a	0.9910
H3–IR	0.16 ± 0.02 a	0.09 ± 0.02 f	206.03 ± 28.33 a	1.36 ± 0.13 b	0.29 ± 0.11 a	0.9919
M1–IR	0.10 ± 0.01 b	0.27 ± 0.03 c	204.17 ± 41.19 a	2.94 ± 0.99 ab	0.44 ± 0.23 a	0.9768
M2–IR	0.05 ± 0.01 cd	0.51 ± 0.03 a	289.61 ± 33.64 a	2.42 ± 0.99 ab	0.40 ± 0.25 a	0.9902
M3–IR	0.07 ± 0.01 bc	0.17 ± 0.01 de	249.58 ± 49.27 a	1.79 ± 0.4686 b	0.39 ± 0.18 a	0.9912
L1–IR	0.03 ± 0.01 de	0.37 ± 0.03 b	206.13 ± 29.83 a	1.70 ± 0.60 ab	0.25 ± 0.17 a	0.9911
L2–IR	0.04 ± 0.01 cde	0.36 ± 0.02 b	277.74 ± 18.73 a	1.62 ± 0.64 b	0.34 ± 0.2 a	0.9992
L3–IR	0.11 ± 0.02 b	0.11 ± 0.02 ef	273.83 ± 25.06 a	1.46 ± 0.03 b	0.36 ± 0.14 a	0.9979

Where H, M, and L represent PPFDs at 550, 350, and 255 μmol·m^−2^·s^−1^, respectively, and numeric values 1, 2, and 3 represent the bottom old leaf, center mature leaf, and the top young expanded leaf, respectively. Data are presented as means ± standard error (n = 5). Different letters represent significant differences among treatments based on Tukey HSD test at a *P* < 0.05 level.

**Table 3:** Results of two-way ANOVA, for photosynthetic parameters and light conditions and leaf position.

Factors		α	*P*n-Max	*I*sat	*I*c	*R*d
**GH**	Light Condition	**	ns	ns	*	ns
	Leaf Position	**	**	*	*	ns
	Light Condition * Leaf Positon	**	ns	ns	ns	ns
**IR**	Light Condition	ns	**	ns	*	ns
	Leaf Position	**	**	*	**	ns
	Light Condition * Leaf Positon	**	**	*	ns	ns

* and ** indicate statistical significance at the 0.05 and 0.01 probability levels (two-way ANOVA), respectively, whereas ns indicate not significant.

**Table 4:** Total chlorophyll concentrations (mg·cm^−2^) of *Anthurium* × ‘Red’ before and after placement in interior rooms.

Leaf Positions	High Light Level		Medium Light Level		Low Light Level	
	Day 0	Day 30	Day 0	Day 30	Day 0	Day 30
Bottom	0.036 ± 0.003 cB	0.037 ± 0.002 cB	0.037 ± 0.003 cB	0.037 ± 0.01 cB	0.045 ± 0.004 bB	0.044 ± 0.005 bB
Central	0.037 ± 0.007 cB	0.045 ± 0.006 bA	0.037 ± 0.003 cB	0.039 ± 0.006 bA	0.044 ± 0.004 bB	0.047 ± 0.004 aA
Top	0.036 ± 0.007 cB	0.044 ± 0.003 bA	0.038 ± 0.009 cB	0.042 ± 0.003 bA	0.042 ± 0.003 bB	0.047 ± 0.004 aA

Data are presented as means ± standard error (n = 5). Different lowercase letters represent significant differences in chlorophyll contents under different light conditions, and uppercase letters show significant differences between 0 and 30 days in various leaf positions based on Tukey HSD test at *P* < 0.05 level.

**Table 5:** Analysis of variance results of the effect of light conditions and leaf positions on the concentration of chlorophyll of *Anthurium* × ‘Red’ before and after placement in interior rooms.

Effect		df	F
Light Condition	Day 0	2.00	2.57
	Day 30	2.00	0.87
Leaf Position	Day 0	2.00	0.58
	Day 30	2.00	3.77 *
Light Condition * Leaf Position	Day 0	4.00	0.08
	Day 30	4.00	0.65

Note: df: degrees of freedom; *F*-values and significance levels (* *P* < 0.05, and ns: *P* > 0.05).

**Table 6:** Plant morphological differences when grown under a shaded greenhouse and interior conditions.

Treatments	Leaf Number		New Leaf Number		Flower Number		Flower Longevity (d)	
	GH	IR	GH	IR	GH	IR	GH	IR
H	12.4 ± 1.14 a	13.4 ± 1.14 a	1.4 ± 0.55 a	1.0 ± 0.71 a	3.2 ± 0.84 a	3.2 ± 0.84 a	55.6 ± 0.55 a	57.2 ± 0.84 a
M	12.8 ± 1.14 a	13.0 ± 0.71 a	1.2 ± 0.45 a	0.6 ± 0.55 a	2.0 ± 0.71 a	2.6 ± 0.55 a	52.8 ± 0.83 b	56.0 ± 0.71 a
L	13.0 ± 1.34 a	12.4 ± 1.41 a	1.0 ± 0.71 a	0.6 ± 0.55 a	2.0 ± 0.00 a	2.4 ± 0.55 a	40.0 ± 1.58 c	44.0 ± 0.58 b

Where GH: greenhouse and IR: interior room; H, M, and L represent PPFDs at 550, 350, and 255 μmol·m^−2^·s^−1^, respectively. Data are presented as means ± standard error (n = 5). Different letters represent significant differences among light levels based on Tukey HSD test at *P* < 0.05 level.

## Data Availability

The data presented in this study are available in the article.
